# Gallbladder Stones in Pediatric Age: An Emerging Problem: The Risk of Difficult Cholecystectomy and the Importance of a Preoperative Evaluation

**DOI:** 10.3390/children10091544

**Published:** 2023-09-13

**Authors:** Camilla Todesco, Francesco Molinaro, Francesca Nascimben, Gianluca Gentilucci, Mario Messina, Andrea Cortese, Vito Briganti, Stefano Tursini

**Affiliations:** 1 Operative Unit of Pediatric Surgery—Azienda Ospedaliera San Camillo Forlanini, Circonvallazione Gianicolense, 87, 00152 Roma, Italyvbriganti@scamilloforlanini.rm.it (V.B.); 2 Department of Medical Sciences, Surgery and Neuroscience, Section of Pediatric Surgery, Policlinico Le Scotte, University of Siena, 53100 Siena, Italy; francesco.molinaro@unisi.it (F.M.); francescanascimben@gmail.com (F.N.); mario.messina@unisi.it (M.M.); 3 Operative Unit of Radiology and Diagnostic Imaging—Azienda Ospedaliera San Camillo Forlanini, 00152 Roma, Italy

**Keywords:** cholelithiasis, choledocholithiasis, cholecystectomy, laparoscopic cholecystectomy, ultrasound, magnetic resonance cholangiography, endoscopic retrograde cholangio-pancreatography, children, pediatric

## Abstract

The need for cholecystectomy during pediatric age has significantly increased in the last two decades. As biliary pathology increases, the probability of complicated cholecystectomies increases too. The aim of this article is to analyze our experience with difficult laparoscopic pediatric cholecystectomy, focusing on the importance of an accurate pre-operative imaging study. We retrospectively analyzed all patients affected by cholelithiasis who underwent laparoscopic cholecystectomy at the Pediatric Surgery Department of San Camillo Forlanini hospital of Rome and Santa Maria alle Scotte University Hospital of Siena from 2017 to 2022. Demographic data, body mass index (BMI), recovery data, laboratory tests, imaging exams, surgical findings, post operative management and outcome were taken into account. Overall, 34 pediatric patients, with a mean age of 14.1 years (6–18 years) were included, with a mean BMI of 29. All patients underwent abdominal ultrasonography and a liver MRI with cholangiography (cMRI). We identified five cases as “difficult cholecystectomies”. Two subtotal cholecystectomies were performed. Cholecystectomy in pediatric surgery can be difficult. The surgeon must be able to find alternative strategies to total cholecystectomy to avoid the risk of possible bile duct injury (BDI). Pre-operative imaging study trough ultrasound and especially cMRI is crucial to recognize possible difficulties and to plan the surgery.

## 1. Introduction

Gallstones are a relatively rare condition in pediatric age [1]. However, in recent years, the prevalence of pediatric cholelithiasis has significantly risen [1,2]. The most recent data available in the literature, even taking into account the wide methodological variability of the available studies, show a prevalence of gallstones in the pediatric age group of between 1.9% and 4% [3].

Risk factors for developing cholelithiasis are traditionally different between adult and pediatric populations. Prematurity, hemolytic diseases, and parenteral nutrition used to often be the primary causes in children [4]. Nevertheless, over the past few years, a significantly higher incidence of cholelithiasis has been observed in obese and diabetic children, as it commonly happens to adults. Unfortunately, the incidence of childhood obesity and diabetes currently is becoming alarming [5]. Cholelithiasis is also being detected more frequently than in previous years, probably due to the increased use of ultrasound, especially in the diagnosis of recurrent abdominal pain [4,5].

As the incidence of biliary pathology rises, so does the request for cholecystectomy and consequently the number of possible complicated interventions [4,6]. Currently, laparoscopy is the technique of choice for performing cholecystectomy: less pain, quicker recovery, better aesthetic results are the well-known advantages associated with this minimally invasive approach [7]. After its introduction, concerns arose over increased rates in bile duct injury (BDI), and thus the concept of the critical view of safety (CVS) has been proposed [8]. The CVS is an anatomical identification technique that aims primarily at identifying the cystic duct and artery [8]. It is part of a larger concept, together with the six-step program of The Society of American Gastrointestinal and Endoscopic Surgeons (SAGES): the Culture of Safety in Cholecystectomy (COSIC), which imposes safety first. This also requires understanding aberrant anatomy and, when needed, to complete the operation with a safe method other than total cholecystectomy [8].

For this reason, a careful pre-operative evaluation is necessary to minimize the risk of complications. It is good practice to perform a detailed radiologic study of the gallbladder and biliary tract. This allows us to highlight any abnormalities in the development of the biliary system as well as assess the presence of stones in the biliary tract, thus making a proper surgical plan.

The aim of this article is to analyze our experience with difficult laparoscopic pediatric cholecystectomy, focusing on the importance of an accurate pre-operative imaging study, using ultrasound (US) and cholangiopancreatography-MRI (cMRI).

## 2. Materials and Methods

This is a retrospective multicenter study. All patients affected by cholelithiasis and/or choledocholithiasis who have undergone laparoscopic cholecystectomy at the Pediatric Surgery Department of San Camillo Forlanini hospital of Rome and Santa Maria alle Scotte University Hospital of Siena from 2017 to 2022 were included.

The retrospective analysis was conducted by consulting the medical records. The diagnosis of cholelithiasis was made through the analysis of anamnestic and clinical data, laboratory exams, and radiological imaging: ultrasound (US) and cholangiopancreatography-MRI (cMRI). cMRI was performed with a GE 1.5 T Voyager machine with Dotarem (gadoteric acid) as a contrast medium. COR T2 TSE; AX T2 TSE; AX T1 GE were analyzed as morphological sequences and radial T2 TSE; MRCP 3D as cholangiography sequences.

For all the patients, we evaluated demographic data, including age at surgery, gender, Body Mass Index (BMI); recovery data, including clinical data (symptoms at diagnosis); lab tests (hemolytic screening, inflammatory markers and liver function tests); imaging exams (US and cMRI); surgical findings (type of operation performed, conversion, elective or emergent), post-operative management (time of stay, short and long term complications), and outcome (clinical recurrences and radiological evaluation). Difficult cholecystectomies were identified, taking into account the definition of “multiple technical intraoperative difficulties that increase the risk for intraoperative and postoperative complications and that prolong the operative time significantly, and may potentially place the patient at significant risk” [9]. The presence of any comorbidities was noted. In all cases, a traditional four trocars “French position” laparoscopic cholecystectomy was performed.

All patients underwent a clinical and US postoperative follow up.

## 3. Results

From January 2017 to May 2022, 34 pediatric patients, 26 females (76.5%) and 8 males (23.5%), with a mean age of 14.1 years (6–18 years) were evaluated at the pediatric emergency department (DEA) of San Camillo Forlanini hospital and University Hospital of Siena because of abdominal pain and then studied at the radiology emergency department for clinical suspicion of gallbladder lithiasis.

The calculated BMI showed a condition of overweight or obesity in all patients apart from two cases of Rett syndrome, which were normal-weight, with a mean BMI of 29 (20–34). In 20 cases (58.8%), the diagnosis of cholecystitis followed an episode of acute localized abdominal pain; in 13 cases (38.2%), the diagnosis was accidental, secondary to a screening for recurrent non-specific abdominal pain; while in 1 case (2.9%), diagnosis followed an episode of pancreatitis. Two patients (5.9%) suffered from Rett Syndrome and one (2.9%) from epilepsy. No hematological pathology or previous parenteral nutrition therapy has been reported in their clinical history.

All patients underwent at least one abdominal ultrasonography, which was sufficient and essential to confirm the clinically suspected diagnosis, showing specific findings of biliary pathology. Additionally, all of them were subjected to a liver MRI with cholangiography (cMRI) for a precise evaluation of the anatomy of the biliary tree and for planning the surgery. No general anesthesia or sedation was needed to perform the II level imaging exams. Four patients (11.7%) underwent II level invasive diagnostic imaging exams; specifically, Endoscopic Retrograde Cholangio-Pancreatography (ERCP) was performed to remove choledochal stones followed by cholecystectomy.

No emergency cholecystectomies were performed.

The mean operating time was 121 min (70–400). None of the laparoscopic cholecystectomies had to be converted to open surgery, with a conversion rate of 0%.

No major or minor intra-operative complications were described, but one (2.9%) peri-operative complication occurred (hemoperitoneum, due to minor bleeding from gallbladder bed) and it needed a re-do surgery. In 29 (85.2%) patients, an abdominal drainage was placed and removed after a mean of 2 days (1–4). Oral feeding was allowed starting from post-operative day one and it was well tolerated in all patients. All patients surgically treated underwent antibiotic therapy for a mean time of 4 days (3–8). The mean total hospital stay was 6.4 days (4–16 days). The mean follow up was 12 months (9–24).

Among the thirty-four cases of laparoscopic cholecystectomies included in the study, we identified five cases as “difficult cholecystectomies” (14.7%), three females (60%) and two males (40%) with a mean age at surgery of 14.5 years (8–18 years). The mean BMI of patients who underwent difficult cholecystectomies was 27, considering though that there were two cases of Rett syndrome of which BMI was on range. Demographic, clinical, laboratory and radiological data of five cases of difficult cholecystectomy are reported in Table 1.

**Figure 1:**
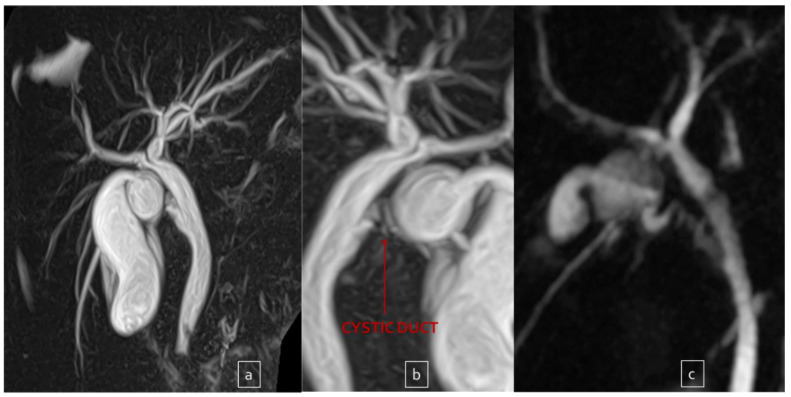
Dysmorphic and dilated infundibulum ((**a**,**b**) front and back view) in a patient with recurrent cholecystitis. And results after partial cholecystectomy (**c**).

**Figure 2:**
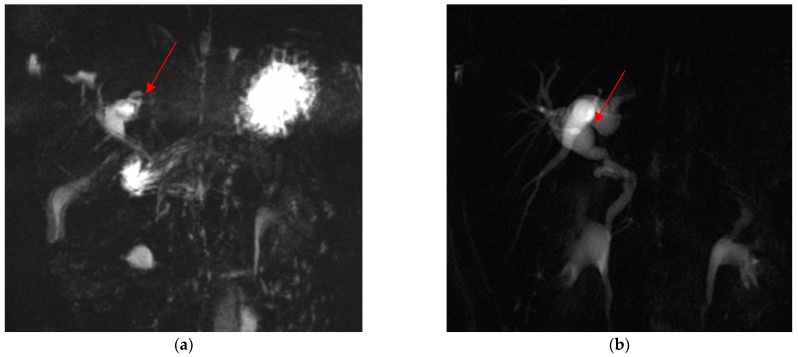
Dysmorphic and dilated left hepatic duct (red arrow) in pre- (**a**) and post-operative (**b**) cMRI.

**Figure 3:**
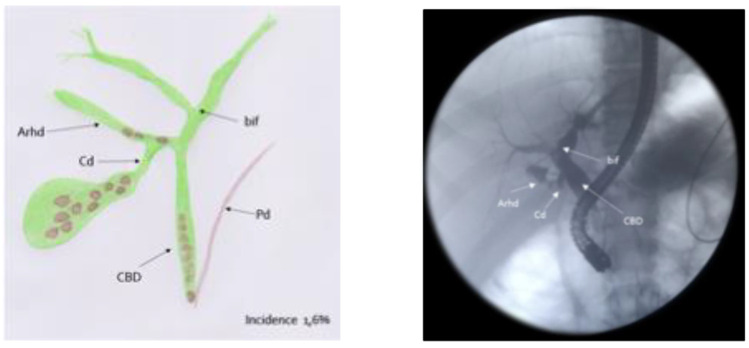
Biliary tract anomaly: the cystic duct projected into an aberrant right anterior hepatic duct and together they flowed into the common bile duct.

Out of these five difficult cases, laparoscopic cholecystectomy was totally completed in three cases (66.7%) and a subtotal cholecystectomy was performed in the other two (33.3%). There was no conversion to open surgery. Surgical difficulty was due to dysmorphic inflamed gallbladder with a dilated infundibulum and a tiny, convolute cystic duct (Figure 1a–c) in one case (20%); dense adhesions at the triangle of Calot due to the chronic inflammation in another case (20%) (Figure 2); an anomaly in the biliary tract anatomy in which the cystic duct projected into an aberrant right anterior hepatic duct and together they flowed into the common bile duct (20%) (Figure 3); and difficult trocar placement and reduced camera work due to the scoliotic attitude of two patients affected by Rett Syndrome (40%) (Table 2). None of these complex cases had Mirizzi syndrome or cholecystoenteric fistulas.

The post-operative course was regular for all patients except for the one with hemoperitoneum. At follow up, patients were all in good general conditions.

## 4. Discussion

Unlike in adults, cholelithiasis has always been considered a relatively rare condition in pediatric patients. Nonetheless, in the last two decades many countries have observed an increase in the incidence of pediatric gallstones and cholecystectomies [2]. Pogorelic et al. show a three-fold increase in the number of cholecystectomy for gallbladder stones in the last ten years compared to the previous ten years period [7]. Similarly, Walker and colleagues found a rise of 213% over a 9-year period [10].

Several studies have tried to correlate this increased incidence with an etiological factor. It has been found that that, just as in adults, BMI plays an important role in the development of cholelithiasis in children [11,12,13,14]. More specifically, patients with cholelithiasis have a significantly higher BMI than the general population and patients with choledocholithiasis have a significantly higher BMI than those with simple stones [5].

As shown in the Results, in our study, almost all patients were overweight, with a clear prevalence in females at 76.5% and in adolescents, with a median age of 14.1 years and a median BMI of 29. In their study, Bailey and colleagues reported a 55% female rate in pediatric patients in 1989. More recently, Mehta and colleagues reported this rate to be 73% [3,15]. It has therefore been suggested that the traditional surgical aphorism for the causes of gallstones should probably now be re-phrased as “female, fat and fifteen” [6].

Other possible causes of cholelithiasis in children are hemolytic diseases, prematurity, total parenteral nutrition use, sepsis, chronic liver disease, inflammatory bowel disease, previous cardiac or abdominal surgery, cystic fibrosis, genetic disposition (progressive familial intrahepatic cholestasis, cystic fibrosis), and diuretic and ceftriaxone use [16]. None of our patients fell into these cases, but two girls were affected by Rett Syndrome, which is an X-linked neurodevelopmental disorder caused by a mutation of the MECP2 gene. It is interesting to notice that the MeCP2 protein seems to be somehow involved in abnormal cholesterol metabolism leading to possible gallstone disease [17].

As mentioned above, as biliary pathology cases increase in number, so does the request for cholecystectomy (which currently are purely laparoscopic) and this could lead to a rise in the so-called “difficult cholecystectomies”. This term refers to “multiple technical intraoperative difficulties that increase the risk for intraoperative and postoperative complications and that prolong the operative time significantly, and may potentially place the patient at significant risk” [9]. No literature is available for the pediatric population. The most common causes can be summarized as severe inflammation that distorts the local anatomy and makes dissections more difficult, and anomalies of the biliary tract [9].

In our series, it was actually due to acute and chronic inflammation and anomaly of the biliary tract but also to a difficulty in positioning trocars and in manipulating the gallbladder with a bad exposition of the Calot triangle in the two severely scoliotic patients affected by Rett Syndrome.

Nassar et al. [18] introduced a scale to classify gallbladders based on gallbladder status, cystic pedicle and degree of adhesions. This scale stratifies cholecystectomies into 4 grades, with grades 3 and 4 being the most difficult and dangerous in terms of possible bile duct injury. To avoid this, Strasberg described CVS in 1995 [19], a rigorous method that requires clarifying the hepatocystic triangle so as to expose the cystic duct, cystic artery, and the cystic plate, in order to prevent major complications such as BDI and damage of the right hepatic artery.

Nevertheless, CVS is not always easy to achieve. Therefore, some authors propose the use of Indocyanine green fluorescence technology (ICG). ICG is secreted entirely from the liver into the bile, so visualization of the biliary tree and particularly of the Calot’s triangle could be a very useful application [20]. Esposito et al. found an ICG sensitivity of 100% in the identification of the gallbladder and the biliary anatomy, irrespectively of the presence of abundant fatty tissue or severe inflammation and adhesions [21].

CVS is only one part of COSIC (culture of safety in cholecystectomy). One of the statements of this culture is “safety first”, and a reliable bailout operation is a key component of safety.

**Figure 4:**
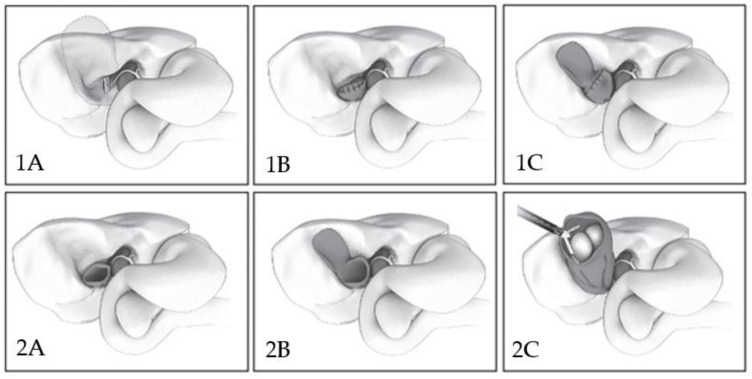
Laparoscopic subtotal cholecystectomy (LSC) classification scheme. Type 1A: normal laparoscopic cholecystectomy. Type 1B: the back wall of the gallbladder (GB) is fully dissected off the liver bed and Hartmann’s pouch is closed following evacuation of gallstones. Type 1C: the back wall of the GB is left in situ and Hartmann’s pouch is closed. Type 2A: the back wall of the GB is excised but Hartmann’s pouch is left open. Type 2B LSC: the back wall of the GB is left in situ and Hartmann’s pouch is left open. Type 2C LSC: the fundus of the GB is deroofed, the stones are evacuated and the GB remnant is left open. These illustrations were created by the Toronto Video Atlas of Surgery (www.tvasurg.ca (accessed on 5 September 2023)) [18].

Surgeons must realize when conditions are too dangerous to proceed in the usual manner and switch to a different approach. Many damage control procedures have been proposed: subtotal cholecystectomy (SC) is one of these. A classification scheme was proposed by Purzner et al. (Figure 4) where the SC were stratified according to whether the posterior wall was left in place or not and whether the remaining part of the gallbladder was closed or not [22]. Regarding complications, a meta-analysis of subtotal cholecystectomy conducted by Elshaer et al. reported a more frequent incidence of bile leakage and sub-hepatic collections when compared to total cholecystectomy [23]. However, in general, complication rates for total cholecystectomy and SC are similar, meaning that difficult GB cases undergoing SC are managed as safely as simple cholecystectomies undergoing TC.

In our two cases, we performed a partial cholecystectomy Type 1A: the back wall of the gallbladder (GB) is fully dissected off the liver bed and Hartmann’s pouch is closed following the evacuation of gallstones. No complications in the post-operative period were observed.

Another possible lifejacket is proposed by Kharytaniuk et al.: laparoscopic aspiration of the gallbladder [24]. Aspiration draws on the principle of “ubi pus, ibi evacua”. In fact, the content of the gallbladder is aspirated via a laparoscopically direct puncture and an antiobiotic solution is then infused. The major advantages of this technique are speed, reproducibility (even by trainees), and intraoperative safety [24]. However, the need for a second look and thus the need for a second general anesthesia make it unattractive for the pediatric population.

We believe that a careful preoperative evaluation is fundamental to make a reasonable surgical plan and to minimize the risk of possible BDI. For this reason, all of our patients underwent not only a preoperative ultrasound, but also a cMRI. cMRI has a sensitivity between 85–92% and specificity of 93–97% for choledocholithiasis. In addition it is a harmless and non-invasive method of bile duct imaging [25] and it does not need general anesthesia. It allows a complete anatomic description of the biliary tree, facilitating an accurate operative strategy. In particularly difficult situations, the ICG technology becomes even more useful.

In conclusion, cholecystectomy in pediatric patients—as it is in adults—can be difficult to perform and its complications could be extremely dangerous. The surgeon must be aware of the risks and be able to find alternative strategies to total cholecystectomy when the latter is impossible. The possibility of a difficult cholecystectomy could be detected in advance by a careful radiological evaluation, especially through the use of cMRI.

## Figures and Tables

**Table 1:** Demographic, clinical, laboratory and radiological data of five cases of difficult cholecystectomy.

Characteristics	Case 1	Case 2	Case 3	Case 4	Case 5
Gender	F	M	M	F	F
Age (years)	15	8	15	17	18
BMI (kg/m^3^)	29.6	28.3	29.4	19	28.4
Associated anomalies	/	/	/	Rett Syndrome	Rett Syndrome
History	Recurrent abdominal pain	Recurrent abdominal pain	/	Chronic diffuse abdominal pain	Chronic non specific abdominal pain
Symptoms	Acute localized abdominal pain, fever	Acute localized abdominal pain	Acute localized abdominal pain	Acute non specific abdominal pain	Acute diffuse abdominal pain
Abdominal US	Acute cholecystits, gallstones, dilatation of biliary tree	Acute cholecystits, gallstones	Gallbladder stones with dilatation of biliary tree	Numerous gall stones without biliary tract dilatation	Numerous gallbladder stones without biliary tract dilatation
Cholangio-MRI	Dysmorphic inflamed gallbladder with tiny infundibulum and convoluted cystic duct	Presence of gallstones	Anomaly in the biliary tract anatomy: cystic duct projected into an aberrant right anterior hepatic duct	Presence of gallstones	Presence of gallstones
ERCP	Removing of two small stoned	Not performed	Removing of numerous small stones in the two hepatic ducts	Not performed	Not performed
VLS cholecystectomy	Subtotal	Total	Subtotal	Total	Total
Post operative US	No biliary dilatation	Persistence of dilated biliary tree	No biliary dilatation	No biliary dilatation	No biliary dilatation
Post operative c-MRI	Persistence of dilated portion of the infundibulum and cystic duct	Not performed	Not performed	Not performed	Not performed
Last follow up (months)	12	10	12	12	12

**Table 2:** Causes of difficult cholecystectomy.

Problems Encountered	N Patients Treated	Conversion Rate	PO Complication Rate
Acute inflammation of the gallbladder	1	0%	0%
Chronic inflammation	1	0%	0%
Difficult trocar placement and reduced camera	2	0%	0%
Anatomical anomalies	1	0%	0%

## Data Availability

Not applicable.
